# Proteinuria in Dent disease: a review of the literature

**DOI:** 10.1007/s00467-016-3499-x

**Published:** 2016-10-18

**Authors:** Youri van Berkel, Michael Ludwig, Joanna A. E. van Wijk, Arend Bökenkamp

**Affiliations:** 1 Department of Pediatric Nephrology, VU University Medical Center, De Boelelaan 1117, 1081 HV Amsterdam, The Netherlands; 2 Department of Clinical Chemistry and Clinical Pharmacology, University of Bonn, Bonn, Germany

**Keywords:** Dent disease, CLCN5, OCRL, Proteinuria, Nephrotic syndrome, Low-molecular weight proteinuria, Systematic review

## Abstract

**Background:**

Dent disease is a rare X-linked recessive proximal tubulopathy caused by mutations in *CLCN5* (Dent-1) or *OCRL* (Dent-2). As a rule, total protein excretion (TPE) is low in tubular proteinuria compared with glomerular disease. Several authors have reported nephrotic-range proteinuria (NP) and glomerulosclerosis in Dent disease. Therefore, we aimed to analyze protein excretion in patients with documented *CLCN5* or *OCRL* mutations in a systematic literature review.

**Design:**

PubMed and Embase were searched for cases with documented *CLCN5 * or *OCRL* mutations and (semi-)quantitative data on protein excretion. The most reliable data (i.e., TPE > protein–creatinine ratio > Albustix) was used for NP classification.

**Results:**

Data were available on 148 patients from 47 reports: 126 had a *CLCN5* and 22 an *OCRL*mutation. TPE was not significantly different between both forms (*p* = 0.11). Fifty-five of 126 (43.7 %) Dent-1 vs 13/22 (59.1 %) Dent-2 patients met the definition of NP (*p* = 0.25). Serum albumin was normal in all reported cases (24/148). Glomerulosclerosis was noted in 20/32 kidney biopsies and was strongly related to tubulointerstitial fibrosis, but not to kidney function or proteinuria.

**Conclusion:**

More than half of the patients with both forms of Dent disease have NP, and the presence of low molecular weight proteinuria in a patient with NP in the absence of edema and hypoalbuminemia should prompt genetic testing. Even with normal renal function, glomerulosclerosis and tubulointerstitial fibrosis are present in Dent disease. The role of proteinuria in the course of the disease needs to be examined further in longitudinal studies.

## Introduction

Dent disease, a rare X-linked recessive tubulopathy, was first described in 1964 in two unrelated cases presenting with renal tubular rickets, hypercalciuria, and tubular proteinuria [1]. Dent disease is characterized by low molecular weight (LMW) proteinuria, hypercalciuria, nephrolcalcinosis or nephrolithiasis, variable manifestations of proximal tubular dysfunction, and progressive renal failure, ultimately resulting in end-stage renal failure in adulthood [2–5].

In about 60 % of patients with X-linked nephrolithiasis, a mutation in the *CLCN5* gene is detected, whereas in 15 %, the disease is due to a mutation in the *OCRL* gene. For the remaining 25 %, no specific gene defect has been described as yet [6]. Patients with a mutation in *CLCN5* are classified as having Dent disease type 1 (Dent-1; MIM #300009), while patients with a mutation in *OCRL* are classified as Dent disease type 2 (Dent-2; MIM #300555). The renal phenotype of Dent-2 is comparable with that of Dent-1 except for a lower prevalence of nephrocalcinosis [7, 8]. The exact prevalence of Dent disease is unknown; to date, >250 families have been described [9].

**Fig. 1:**
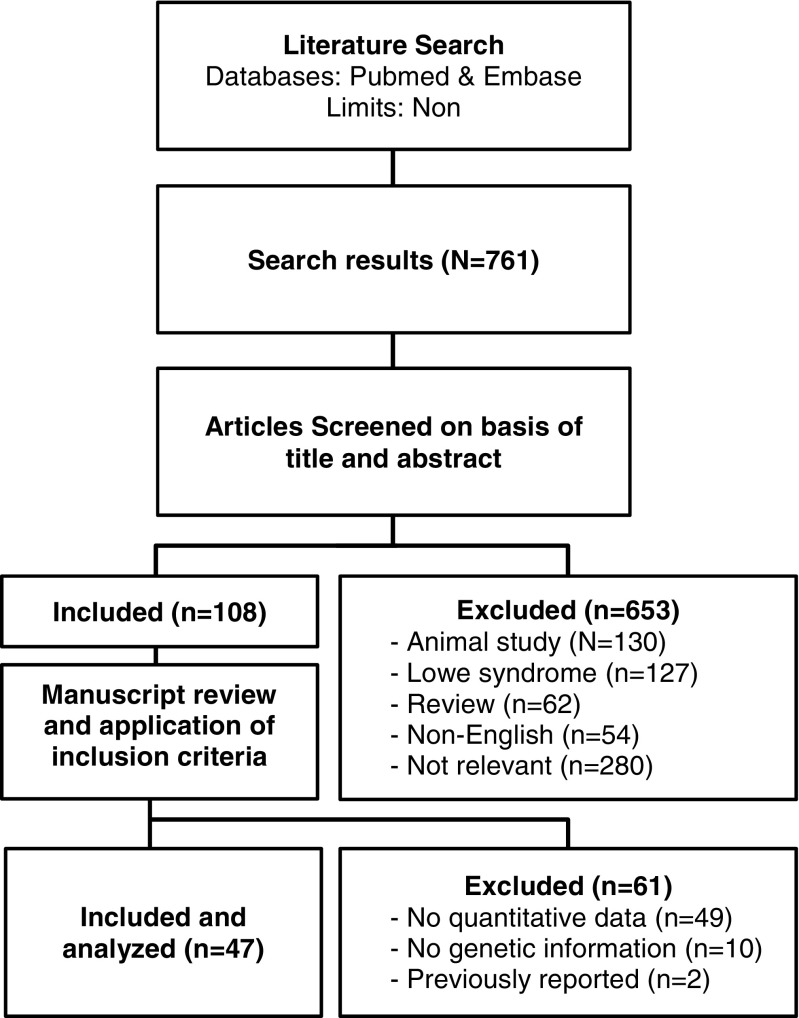
Study flow

Under normal conditions, LMW proteins, which pass the glomerular membrane extensively [10], are almost completely reabsorbed in the proximal tubule by megalin-cubilin receptor-mediated endocytosis and therefore do not appear in the final urine. As *CLCN5* and *OCRL* mutations impair the function of the megalin–cubilin system, the loss of LMW proteins is an obligate finding in Dent disease [11]. As a rule, total protein excretion is low in tubular proteinuria compared with glomerular disease, mostly around 1 g/l, and rarely exceeds 2 g/l [12]. Frishberg et al. [2], Copelovitch et al. [13], and Fervenza [14] reported nephrotic-range proteinuria (NP) in patients with documented *CLCN5* mutations and histological findings of focal segmental glomerulosclerosis (FSGS) or focal global glomerulosclerosis. They suggested that Dent-1 disease is an underdiagnosed etiology of FSGS and might reflect a yet unrecognized glomerular dysfunction in Dent-1, while data on proteinuria in Dent-2 are sparse [15]. Therefore, the objective of this study was to analyze protein excretion and histology in patients with documented *CLCN5* or *OCRL* mutation disease by means of a systematic literature review (Fig. 1).

## Methods

Our search was performed using the bibliographic search engines PubMed and Embase using the following search strategy:

- For Dent-1: [Dent’s disease OR Dents disease OR Dent disease OR Dent Disease (Mesh)] AND [CLCN5 OR CLCN-5 OR CLCN 5 OR CLCN OR CLC-5 chloride channel (Supplementary Concept) OR Dent disease 1 (Supplementary Concept)]
- For Dent-2: Dent’s disease OR Dents disease OR Dent disease OR Dent Disease (Mesh)] AND [OCRL1 OR OCRL-1 OR OCRL 1 OR OCRL OR Dent disease 2 (Supplementary Concept)].

The combined search on Embase and PubMed yielded a total of 761 unique records, all of which were screened manually by title and abstract by two independent researchers (YvB and AB). Inclusion criteria were papers describing (i) Dent disease patients with documented *CLCN5* or *OCRL* mutations, and (ii) presenting quantitative or semiquantitative data on proteinuria. Main exclusion criteria were cases with extrarenal findings, suggesting Lowe syndrome in *OCRL*-positive patients, and papers reporting female carriers only. Only papers published in English were considered for review. The reference lists of the respective papers were checked for publications missed by the search strategy.

Data on total protein excretion were extracted if presented as (i) semiquantitative Albustix in a spot urine sample (Sticks), (ii) protein–creatinine ratio in a spot urine sample (P/C), or (iii) as protein excretion in a timed urine collection. Timed protein excretion (TPE) was related to total body surface area (BSA) and presented as milligrams per meter^2^ per hour. BSA was calculated from weight and height using the Mosteller formula [16]. In 54 patients with documented timed proteinuria, no height/weight data were available, however. In these cases, BSA was estimated as follows: in adults, a standard BSA of 1.73 m^2^ was used, while in children, height and weight were extrapolated using Dutch reference data of gender- and age-specific medians for height and weight [17]. To validate this approach, we compared TPE based on extrapolated BSA, with TPE calculated from documented height and weight in 28 patients. Median TPE based on extrapolated BSA was 52.1 mg/m^2^/h [interquartile range (IQR) 40.3–71.2] compared with 51.6 mg/m^2^/h with documented height and weight (IQR 35.8–71.6, *p* = 0.699, Wilcoxon test), indicating that our approach yields valid results. If TPE was expressed as milligrams per kilogram per day, it was transformed into milligrams per meter^2^ per hour by analogy.

The following definitions were used: LMW proteinuria was defined as increased excretion of either β_2_ microglobulin (β2M) (>0.3 mg/l or >0.3 mg/g creatinine), retinol-binding protein (RBP) (>0.1 mg/l or >0.1 mg/g creatinine), or α_1_ microglobulin (α1M) (>12 mg/l or >11.7 mg/g creatinine) [18, 19]. NP was defined as TPE > 40 mg/m^2^/h [20] or P/C > 1800 mg/g creatinine (corresponding to >200 mg/mmol creatinine) [21] or Sticks 3+ [21]. If more than one parameter was available, the most reliable (i.e. TPE > P/C > sticks) was used for NP classification. Unless glomerular filtration rate (GFR) was stated in the paper, it was estimated using the Schwartz equation in children [22] or the four variable Modification of Diet in Renal Disease (MDRD) equation in adults [23]. As functional data are lacking for most mutations, mutations leading to messenger RNA (mRNA) decay or premature termination of the resultant OCRL-1 or ClC-5 protein (i.e., stop, frameshift, gross deletion) were classified as severe, while frame and missense mutations were classified as moderate [24].

Serial data were available on only 13 patients. In these cases, data at presentation were used. Statistical analysis was performed using SPSS version 22 [25]. Data are presented as median [interquartile range (IQR)] and were analyzed using nonparametric tests (chi-square and Mann–Whitney* U*). Differences were considered significant at *p* <0.05, and *p* <0.1 was considered as a statistical trend.

## Results

### Study characteristics

As of 1 March 2016, the PubMed and Embase search yielded a total of 761 publications after removing 499 duplicates. Six hundred and fifty-three were excluded based on title and abstract (130 involved animal studies, 127 reported on Lowe syndrome, 62 were reviews, 54 were not in English, and 280 were irrelevant to our study). The remaining 108 articles were reviewed in full text: 61 were excluded (49 presented nonquantitative data, diagnosis of Dent disease had not been confirmed genetically in ten, and two papers contained cases already reported in another publication). In total, 47 articles describing 148 patients were included [2, 5, 13–15, 26–67]. The median number of patients per publication was two (IQR 1–5). Sixty-one patients were reported in papers from Japan, China, Korea, or India; 24 from North America; three from South America; 55 from the Mediterranean; and five from Northern Europe. One hundred and-twenty-six patients had a *CLCN5* and 22 an *OCRL* mutation. Median age was 8.5 years (IQR 4–13) in Dent-1 and 9 years (IQR 4–10.5) in Dent-2. Data on GFR were available in 110 patients with Dent-1 and 19 with Dent-2. Here, too, no significant difference was found [110.5 ml/min/1.73 m^2^ (IQR 79–143) vs. 108 ml/min/1.73 m^2^ (IQR 94–130); *p* = 0.971].

### Proteinuria

Data on proteinuria are presented in Table 1. In the majority of patients, TPE was reported. There was no difference in proteinuria between Dent-1 and Dent-2 patients based on TPE, while Dent-2 patients scored higher when proteinuria was tested by urinary dipstick. In both groups, almost half of the patients had NP. Quantitative LMW proteinuria was reported in 134 patients. β2M was by far the most frequently measured LMW protein and was reported in 85.1 % of patients; the vast majority of papers reported LMW protein concentrations rather than LMW protein/creatinine ratios or TPE. This was also the case for urinary albumin excretion, which was reported in only 11 patients [median 200 mg/l (IQR 80–310)]. This precluded further analysis of urinary protein composition. There was no significant relationship between TPE and GFR (*r* ^2^ = 0.032, *p* = 0.15). Accordingly, GFR was comparable between patients with and without NP [104.1 ml/min/1.73 m^2^ (IQR 79–121.6) vs. 118.3 ml/min/1.73 m^2^ (IQR 83.6–173.9); *p* = 0.327)] There was no single report mentioning edema formation in Dent patients. Serum albumin was reported in 24 cases: median concentration was 46.5 (39–54) g/l.

**Table 1:** Proteinuria in Dent-1 and Dent-2 patients

	Total	Dent-1	Dent-2	*P* value
Dipstick (*n*)	*N* = 63	*N* = 52	*N* = 11	0.02
1+	18 (27.3 %)	17 (30.9 %)	1 (9.1 %)	
2+	40 (60.6 %)	34 (61.8 %)	6 (54.5 %)	
3+	7 (10.6 %)	3 (5.5 %)	4 (36.4 %)	
4+	1 (1.5 %)	1 (1.8 %)	0 (0 %)	
P/C (mg/g)	*N* = 23	*N* = 20	*N* = 3	*NA*
	2800 (2100–4104)	2850 (1835–4900)	2718 (NA)	
TPE (mg/m^2^/h)	*N* = 83	*N* = 71	*N* = 12	0.11
	49.0 (38.1–69.1)	47.0 (37.3–67.4)	57.5 (41.8–81.1)	
NP (*N*/*N*)	68/148 (45.9 %)	55/126 (43.7 %)	13/22 (59.1 %)	0.25

Data presented as number (%) of cases or as median and interquartile range (IQR)

*P/C* protein–creatinine ratio, *TPE* timed protein excretion, *NP* nephrotic-range proteinuria

Information on renin–angiotensin–aldosterone system (RAAS) inhibition was available for eight patients only. Angiotensin-converting enzyme (ACE) inhibitors or angiotensin II receptor blockers (ARB) were prescribed for eight Dent-1 patients (Table 2): all but one had NP; a reduction of proteinuria of 50 % was reported for two; proteinuria remained unchanged in four. In one patient, RAAS inhibition was discontinued because of rapidly rising serum creatinine. In two others, GFR halved within 4 and 7 years, respectively.

**Table 2:** Renin–angiotensin–aldosterone system (RAAS) inhibitor use

Author	Patient ID	Mutation	Age (years)	ACE inhibitor	ARB	T = 0	T = 1	Treatment duration
Copelovitch et al. [13]	Patient 1	*CLCN5*	12	Yes	No	Proteinuria: 50 mg/kg/day	Proteinuria: 25 mg/kg day	2 months
Frishberg et al. [2]	Patient 1	*CLCN5*	9	Yes	Yes	NA	No effect on proteinuria	*NA*
	Patient 3	*CLCN5*	14	Yes	Yes	*NA*	No effect on proteinuria	*NA*
Lim et al. [44]	Case 1	*CLCN5*	3	Yes	No	*NA*	50 % P/C reduction	6 months
Marsenic et al. [47]	Patient 1	*CLCN5*	13	Yes	No	*NA*	Strong rise in serum creatinine	*NA*
Okamoto et al. [52]	Case 1	*CLCN5*	3	Yes	No	GFR 73 ml/min/1.73 m^2^	GFR 30 ml/min/1.73 m^2^	7 years
Vaisbich et al. [63]	Case 1	*CLCN5*	11	Yes	No	Proteinuria: 51 mg/kg day GFR 66 ml/min/1.73 m^2^	Proteinuria: 65 mg/kg day GFR 65 ml/min/1.73 m^2^	3 years 5 months
	Case 2	*CLCN5*	4	Yes	No	Proteinuria: 65 mg/kg day GFR 172 ml/min/1.73 m^2^	Proteinuria: 60 mg/kg day GFR 95 ml/min/1.73 m^2^	4 years

*ARB* angiotensin II receptor-blockers, *ACE* angiotensin-converting enzyme, *GFR* glomerular filtration rate,* T = 0* before therapy,* T = 1* during therapy

### Mutation

In 44 of 148 patients (29.7 %), mutation severity was classified as moderate. In this group, the prevalence of NP was comparable with patients harboring a severe mutation (52.3 % vs 43.3 %, *p* = 0.368), as was proteinuria [TPE 60.0 mg/m^2^/h (IQR 39.9–77.6) vs. 46.1 mg/m^2^/h (IQR 36.5–64.2, *p* = 0.205) and GFR [113.9 ml/min/1.73 m^2^ (IQR 74.4–162.4) vs 108.2 ml/min/1.73 m^2^ (IQR 81–138), *p* = 0.584).

### Kidney biopsy

Kidney biopsy data were reported in 34 patients: 31 Dent-1 and three Dent-2. Proteinuria [TPE 52.1 mg/m^2^/h (IQR 39–69) vs. 48.8 mg/m^2^/h (IQR 37–78), *p* = 0.80) and GFR [118 ml/min/1.73 m^2^ (IQR 83–166) vs. 109 ml/min/1.73 m^2^ (IQR 79–140), *p* = 0.51] were comparable between biopsied and nonbiopsied patients. Median age at biopsy was 9 years, which is identical with the median age of the total cohort. Table 3 summarizes biopsy results. In ten patients, glomeruli were reported to be normal; in two, immature glomeruli were noted; and in two, no information was provided. Glomerular sclerosis was noted in 20 of the 32 biopsies describing glomerular morphology (63 %), namely, focal global glomerulosclerosis (*N* = 18) rather than FSGS (*N* = 5). In patients in whom glomerulosclerosis was quantified, a median of 13 % (IQR 6–26) of glomeruli was affected. Only four papers reported electron microscopy data on glomerular foot processes: in a single patient [44], foot-process effacement was noted; in the other three, electron microscopy was normal [27, 36, 42]. Tubulointerstitial fibrosis was present in 14 of 29 patients (48 %) and nephrocalcinosis in ten of 14 (71 %) in whom these features were reported. Contingency table analysis between glomerular sclerosis and tubulointerstitial fibrosis showed a highly significant association (χ^2^ = 19.1, *p* = 0.001). There was no statistical difference in median TPE with respect to glomerulosclerosis [with 49.1 mg/m^2^/h (IQR 37–78) vs. without 65.8 mg/m^2^/h (IQR 52–69), *p* = 0.1] or tubulointerstitial fibrosis [with 49.1 mg/m^2^/h (IQR 33–84) vs. without 61.8 mg/m^2^/h (IQR 47–68), *p* = 0.238]. Of note, both histological findings appeared to be associated with less rather than more proteinuria.

**Table 3:** Kidney biopsy results

Author	Patient ID	Mutation	Age (years)	Sticks (0–4+)	P/C (mg/g creat)	TPE (mg/m^2^/h)	GFR (ml/min/1.73 m^2^)	Glomeruli in biopsy (*N*)	Sclerosis global [*N* (%)]	Sclerosis focal [*N* (%)]	Tubulointerstitial fibrosis	Calcinosis
Anglani et al. [27]	Case 2	*CLCN5*	15	*NA*	*NA*	23.4	140	*NA*	0	0	+	*NA*
Becker-Cohen et al. [28]	Patient 3	*CLCN5*	10	*NA*	2898	*NA*	83	*NA*	FGS n.o.s.	0	*NA*	*NA*
Cheong et al. [32]	Patient 2	*CLCN5*	9	*NA*	*NA*	45.5	127	*NA*	0	0	−	+
Copelovitch et al. [13]	Patient 1	*CLCN5*	12	*NA*	1602	46.1	76	37	9 (25)	0	+	*NA*
	Patient 2	*CLCN5*	9	*NA*	*NA*	55.2	79	6	2 (33)	2 (33)	+	*NA*
Fervenza et al. [14]	Case 1	*CLCN5*	18	*NA*	*NA*	108.4	44	21	4 (19)	0	+	*NA*
Frishberg et al. [2]	Patient 1	*CLCN5*	9	*NA*	*NA*	42.4	131	50	2 (4)	0	−	*NA*
	Patient 3	*CLCN5*	14	*NA*	*NA*	32.3	122	50	5 (10)	1 (2)	−	*NA*
	Patient 4	*CLCN5*	9	4+	2151	49.1	127	24	2 (8)	2 (8)	+	*NA*
Hellemans et al. [36]	Patient 1	*CLCN5*	20	*NA*	*NA*	73.3	61	*NA*	0	0	−	*NA*
Hoopes et al. [37]	A-4-1	*CLCN5*	4	*NA*	*NA*	34.0	*n.a*	*NA*	FGS n.o.s.	0	+	+
Igarashi et al. [39]	Family 1.3	*CLCN5*	6	2+	*NA*	*NA*	132	*NA*	FGS n.o.s.	0	*NA*	+
	Family 3.8	*CLCN5*	15	2+	*NA*	*NA*	175	*NA*	FGS n.o.s.	0	++	+
	Family 3.9	*CLCN5*	13	2+	*NA*	*NA*	113	*NA*	FGS n.o.s.	0	++	+
	Family 5.13	*CLCN5*	16	1+	*NA*	*NA*	151	*NA*	FGS n.o.s.	0	++	*NA*
Kaneko K et al. [40]	Case	*OCRl*	4	*NA*	*NA*	81.8	194	*NA*	0	FSGS n.o.s.	−	*NA*
Langlois et al. [42]	Case 1	*CLCN5*	7	*NA*	*NA*	24.4	150	*NA*	FGS n.o.s.	0	++	+
	Case 3	*CLCN5*	2	*NA*	*NA*	39.3	84	42	0	2 (5)	++	*NA*
Li et al. [43]	Patient 1	*CLCN5*	11	*NA*	*NA*	24.3	92	*NA*	0	0	−	*+*
Lim et al. [44]	Case 1	*CLCN5*	3	*NA*	*NA*	181.5	135	19	0	0	−	*NA*
Matsuyama et al. [48]	3.II.1	*CLCN5*	8	2+	*NA*	*NA*	182	53	7 (13)	0	−	−
	1.II.1	*CLCN5*	12	2+	*NA*	*NA*	171	45	Immature	Immature	*NA*	+
Morimoto et al. [49]	2-2-c	*CLCN5*	21	2+	*NA*	*NA*	100	*NA*	0	0	−	−
Okamoto et al. [52]	Case 1	*CLCN5*	3	2+	*NA*	59.0	97	*NA*	FGS n.o.s.	0	++	*NA*
Ramos-Trujillo et al. [53]	P274	*CLCN5*	5	*NA*	*NA*	*34.0*	*NA*	*NA*	0	0	−	−
Sekine et al. [55]	Patient 2	*OCRl*	16	3+	*NA*	*NA*	102	*NA*	0	0	−	*NA*
Sheffer-Babila et al. [57]	Patient A	*CLCN5*	8	*NA*	*NA*	87.0	68	18	3 (17)	0	*NA*	*NA*
	Patient B	*CLCN5*	11	*NA*	*NA*	49.0	101	*NA*	FGS n.o.s.	0	*NA*	*NA*
Takemura et al. [58]	Patient 1	*CLCN5*	2	2+	*NA*	69.1	*NA*	*NA*	0	0	−	*NA*
	Patient 2	*CLCN5*	3	2+	*NA*	39.2	*NA*	*NA*	NA	*NA*	−	+
Tasic et al. [59]	Patient 3	*OCRl*	10	*NA*	*2718*	*NA*	*NA*	*NA*	0	*0*	−	−
Vaisbich et al. [63]	Case 1	*CLCN5*	11	*NA*	*NA*	61.8	75	84	23 (27)	*NA*	+	*NA*
	Case 2	*CLCN5*	4	*NA*	*NA*	65.2	193	*NA*	*NA*	*NA*	−	+
Yanagida et al. [64]	Case 1	*CLCN5*	2	2+	*NA*	69.1	*NA*	*NA*	0	0	+	*NA*
Median (IQR)			9 (4–13)	2+	2435	49.1 (35–69)	117 (83–148)	40 (20–50)	17 %	6 %		

*P/C* protein–creatinine ratio, *TPE* timed protein excretion, *GFR* glomerular filtration rate, *FGS* focal global sclerosis, *FSGS* focal segmental glomerulosclerosis, *n.o.s*. not otherwise specified, *NA* not available,* −* absent,* +* mild,* ++* severe

## Discussion

We demonstrated that NP is a common finding in Dent disease both in patients with *CLCN5* and *OCRL* mutations. Still, patients do not present with the other two characteristics of nephrotic syndrome (NS), i.e., edema formation or hypoalbuminemia. Therefore, the finding of isolated NP should prompt analysis for LMW proteinuria. Although LMW proteinuria can be seen as a result of overflow proteinuria in patients with massive proteinuria, this phenomenon is not observed in the proteinuria range of Dent patients [68]. Two mechanisms may be involved in the pathogenesis of NP in Dent disease: defective uptake of physiologically filtered albumin and other proteins, or an additional glomerulopathy as suggested by the presence of glomerulosclerosis on renal biopsy [2, 13, 14].

Although it was held that *CLCN5* is only expressed in proximal tubuli and collecting ducts, Ceol et al. recently showed *CLCN5* expression in human podocytes [69]. As *CLCN5* appeared to be overexpressed in proteinuric states, the authors speculate that podocytes have an endocytic machinery similar to the proximal tubular cell. This has been linked to the finding of glomerulosclerosis in Dent-1 patients [14]. Studies in rabbits showed that *OCRL*, too, is expressed in glomeruli [70]. This prompted us to compare the proteinuric phenotype in both forms of Dent disease, which share a common metabolic pathway in the proximal tubule. In line with findings on renal–tubular dysfunction in both patient groups [11], we found that proteinuria was virtually identical, suggesting defective protein uptake as the underlying mechanism.

Can defective protein uptake by the megalin–cubilin complex alone account for the amount of proteinuria observed in these patients? While LMW proteins are almost freely filtered across the glomerular membrane, there is much controversy about glomerular albumin filtration, with sieving coefficients ranging from 0.0006 to 0.074 in different rat models [71]. This has recently been reviewed by Dickson et al., who suggest that glomerular albumin filtration is higher than previously thought and that part of the filtered albumin is reabsorbed intact via transcytosis involving the neonatal Fc receptor [72]. Norden et al. studied patients with Dent disease to calculate the glomerular sieving coefficients of albumin, immunoglobulin (Ig) G and other plasma proteins in vivo, assuming that in these patients, urinary protein excretion directly reflects glomerular protein filtration [10]. They report a sieving coefficient for albumin of 0.00008 and a mean urine albumin/creatinine ratio of 38 mg/mmol (i.e. 342 mg/g creatinine).

In another study on tubular proteinuria in Dent disease, Norden et al. reported a linear relationship between the albumin/creatinine ratio and the creatinine-corrected concentrations of retinol-binding protein, β2M, and alpha-1 microglobulin, all of which were around 20–30 mg/mmol creatinine (i.e., 180–270 mg/g) [18]. The sum of the mean albumin, retinol-binding protein, β2M, and alpha-1 microglobulin concentrations standardized for urine creatinine was 100 mg/mmol (i.e., 900 mg/g), which is about one third of the proteinuria observed in our study. This probably reflects the multitude of other LMW proteins and polypeptides undergoing tubular reabsorption, which were not measured in their study. Of note, intermittent nephrotic-range proteinuria has also been observed in a pair of siblings with a mutation in *CUBN* encoding for cubilin, whereas most of these patients only have LMW proteinuria [73].

Kidney biopsy data were only available for ∼20 % of patients and might be flawed by a reporting bias. Still, patient age, renal function, and amount of proteinuria were identical to those of nonbiopsied patients. When reported, the number of glomeruli was sufficient to exclude a serious sampling error. The finding of glomerulosclerosis in almost two thirds of the biopsies (with a median of 17 % glomeruli being sclerosed) underscores the concerns about glomerular pathology in Dent disease. However, glomerulosclerosis was mostly focal global rather than focal segmental [2, 13, 14]. The absence of foot-process effacement (when reported) and the missing association between the amount of proteinuria and tubulointerstitial fibrosis or the presence of glomerulosclerosis, points toward a primary tubulointerstitial process leading to glomerular sclerosis. This fits with the strong association between glomerulosclerosis and tubulointerstitial fibrosis, which was also found by Theilig et al. in megalin-knockout mice [74]. In their model, megalin deficiency was associated with increased apoptosis at baseline, which was accentuated following induction of anti-glomerular-basement-membrane (GBM) nephritis. Recent studies in a model of isolated proximal tubular damage have shown that tubulointerstitial injury can lead to glomerulosclerosis [75, 76].

Reports regarding RAAS inhibition in Dent disease are scarce, and most of them report disappointing results. RAAS inhibition is the treatment of choice for glomerular proteinuria originating either from glomerulonephritis, Alport syndrome, or primary or secondary FSGS. Based on the strong association between urinary albumin excretion and renal endpoints, Heerspink and Gansevoort argue that normalization of urinary albumin excretion should be a therapeutic target per se [77]. They show that the beneficial effect of RAAS inhibition is conveyed via normalization of albuminuria. Does this also apply to albuminuria resulting from defective tubular reabsorption? There is a large body of evidence mostly from in vitro studies indicating that an overload of albumin in primary urine triggers a toxic effect and inflammatory response in proximal tubular cells conveyed via the megalin–cubilin system [77]. In their study on anti-GBM disease in megalin-knockout mice, Theilig et al. indeed observed increased expression of profibrotic markers in a megalin-dependent manner [74]. In this light, disturbed endocytosis—as in Dent disease—might be expected to protect the proximal tubular cells against the detrimental effects of albuminuria. However, tubulointerstitial fibrosis was most marked in knockout animals, leading the authors to challenge the view of protein overload causing tubulointerstitial damage. Norden et al. demonstrated tubular wasting of a wide variety of polypeptides, hormones (e.g., insulin, growth hormone, insulin-like growth factor-1), and chemokines (e.g., monocyte chemoattractant protein-1) [10]. Some of these have been implicated in the pathogenesis of tubulointerstitial fibrosis and may be the underlying mechanism for progressive renal failure in Dent disease.

Due to the nature of a review based on case reports, our study has several limitations: (i) The number of reported Dent-2 patients is far less than that of Dent-1 patients; therefore, nonparametric tests had to be used, which may have weakened the statistical power of our analysis. Still, the findings in both patient groups were virtually identical, indicating that our conclusion that Dent-1 and Dent-2 are phenotypically identical as far as proteinuria is concerned holds true. (ii) Data were analyzed retrospectively, and data sets were incomplete for many parameters. In particular, for TPE, assumptions were made regarding BSA based on Dutch reference values, while the literature survey identified mostly patients from Asia and the Mediterranean. As the Dutch are taller on average than these populations, BSA has probably been overestimated in our analysis. Therefore, TPE will be even higher than estimated. The lower prevalence of NP by dipstick analysis compared with quantitative methods reflects the limitation of this test for detecting LMW proteinuria. Data on albuminuria was scarce, precluding a specific analysis of protein composition. (iii) In particular, in patients with high protein excretion, a publication bias cannot be excluded. However, the majority of reports did not focus on proteinuria and did not even discuss the amount of protein excretion, indicating that our findings on median protein excretion are representative for Dent patients. (iv) Patients were rather young in our series and had normal GFR. Also, there was insufficient data for serial analysis, and thus our data cannot be extrapolated to older Dent patients, and the prognostic role of proteinuria in Dent disease remains unclear. Here, prospective long-term studies are needed to evaluate the impact of proteinuria and hypercalciuria/nephrocalcinosis for the prognosis of Dent disease. Such studies are underway (http://www.rarekidneystones.org). Potential therapeutic interventions to be tested are thiazide diuretics, citrate, and RAAS inhibition [3]. Regardless, the slow natural course of Dent disease leading to end-stage renal disease in later adulthood is a major obstacle for prospective (interventional) studies. Animal studies such as the citrate trial in knockout mice performed by Cebotaru et al. [78] may be more informative than studies in humans using surrogate markers such as hypercalciuria [79] or proteinuria.

## Conclusion

Nephrotic-range proteinuria is a common finding both in Dent-1 and Dent-2 and reflects disturbed protein reabsorption in the proximal tubule rather than glomerular damage. The presence of LMW proteinuria is the hallmark of Dent disease and should be tested in patients with nephrotic-range proteinuria without edema and hypoalbuminemia in order to avoid potentially harmful immunosuppressive therapy and unnecessary renal biopsies. Even with normal renal function, glomerulosclerosis and tubulointerstitial fibrosis are already present in the majority of patients in childhood. The role of proteinuria in the course of the disease needs to be studied further in longitudinal (interventional) studies.
